# Haemoplasma Prevalence and Diversity in Three Invasive *Rattus* Species from Gauteng Province, South Africa

**DOI:** 10.3390/microorganisms10081632

**Published:** 2022-08-12

**Authors:** Liezl Retief, Christian T. Chimimba, Marinda C. Oosthuizen, Asiashu Matshotshi, Armanda D. S. Bastos

**Affiliations:** 1 Department of Zoology and Entomology, Mammal Research Institute (MRI), University of Pretoria, Hatfield 0028, South Africa; 2 DSI-NRF Centre of Excellence for Invasion Biology (CIB), Department of Zoology and Entomology, University of Pretoria, Hatfield 0028, South Africa; 3 Department of Veterinary Tropical Diseases, Faculty of Veterinary Science, University of Pretoria, Hatfield 0028, South Africa; 4 Department of Life and Consumer Sciences, College of Agriculture and Environmental Sciences, University of South Africa, Pretoria 0002, South Africa

**Keywords:** *Mycoplasma*, haemoplasmas, *Rattus rattus*, *Rattus tanezumi*, *Rattus norvegicus*, phylogeny, 16S rRNA, Rnase P, *dna*K, *gap*A

## Abstract

Invasive *Rattus* species are carriers of haemotropic *Mycoplasmas* (haemoplasmas) globally, but data from Africa are lacking. Using a PCR-sequencing approach, we assessed haemoplasma prevalence and diversity in kidney and buccal swabs collected from three invasive *Rattus* species (*Rattus rattus*, *R. norvegicus* and *R. tanezumi*) in Gauteng Province, South Africa. Whilst the overall sequence-confirmed haemoplasma prevalence was 38.4%, infection rates in *R. rattus* (58.3%) were significantly higher (χ^2^ = 12.96; df = 2; n = 99 *p* < 0.05) than for *R. tanezumi* (14.3%). Differences between host sex (χ^2^ = 3.59 × 10^−31^; df = 1; n = 99; *p* = 1.00) and age (χ^2^ = 4.28; df = 2; n = 99; *p* = 0.12) were not significant. Whilst buccal (1.01%) and ectoparasite positivity (2.13%) were low, these results suggest that multiple transmission routes are possible. Three phylogenetically distinct lineages, consistent with global rat-associated strains described to date, were detected, namely, ‘*Candidatus* Mycoplasma haemomuris subsp. Ratti’, and two Rattus-specific haemoplasmas that are yet to be formally described. These results expand the known distribution of invasive rat-associated haemoplasmas and highlight the potential for pathogen co-invasion of new territories together with invading rodent hosts.

## 1. Introduction

Haemotropic mycoplasmas or haemoplasmas represent a group of potentially emergent, unculturable bacteria that have garnered interest as the causative agents of infectious anaemia or haemoplasmosis in a wide range of mammalian species [1,2]. Originally classified into two separate genera, *viz. Haemobartonella* and *Eperythrozoon,* within the family Anaplasmataceae, members of this group have been reclassified within the genus *Mycoplasma* based solely on the 16S rRNA gene phylogeny, a decision which remains contentious [3,4,5,6]. This over-reliance on the highly conserved ribosomal gene is due to the difficulties with in vitro culture, low levels of detection associated with microscopic examination of blood smears, and the lack of polymerase chain reaction (PCR) assays with a broad species recognition range [6,7]. As a result, 16S rRNA amplification and sequencing are currently the gold standards for detecting and characterising haemoplasmas [8]. PCR assays targeting the 23S rRNA and the Rnase P gene regions are used to a lesser degree [7,8], and alternative targets such as *gapA, dna*k, and *gyr*B have limited applications as these assays have a narrow species-recognition range. Consequently, 16S rRNA gene sequences currently make up most of the genetic data available for haemoplasmas in public databases [6,9].

The severity of haemoplasma infection varies depending on the haemoplasma species as well as the infected host species and can range from asymptomatic chronic infection to severe acute haemolytic anaemia, which can lead to anorexia, lethargy, dehydration, weight loss, pyrexia, and death [10,11]. Immunocompromised, splenectomised, or, in the case of domestic cats, young individuals as well as individuals co-infected with more than one haemoplasma species or other pathogens often display more severe symptoms [7,12]. In certain mammalian taxa, such as domestic dogs [13] and cats [14] as well as Darwin’s foxes [15], older individuals exhibit a higher haemoplasma prevalence compared to juveniles, likely as a result of haemoplasma infection being enzootic in these hosts [15]. However, as most of these studies on haemoplasma pathology involve mammalian domestic species, information regarding disease severity in free-living species is limited [7]. Similarly, little is known about the intra- and inter-specific transmission routes of haemoplasmas. Haemoplasmas are likely either transmitted through shared bloodsucking arthropods, such as fleas, ticks, and lice, or through direct contact during social or aggressive interactions between infected and uninfected hosts [7]. Some authors have suggested that haemoplasmas likely use more than one transmission route [7,11,16].

Traditionally, research on haemoplasmas has focused on infections of domestic mammalian species, including cats [17], dogs [18], pigs [19], and cattle [20]. However, in the last decade, haemoplasmas have been reported in multiple free-living mammalian species, including rodents [16,21,22,23,24,25], bats [15], felids [26], canids [27], and racoons [28]. Some haemoplasma strains, notably *Mycoplasma haemohominis* (an *M.* *haemofelis*-like strain), *M. ovis* and “*Candidatus* Mycoplasma haematoparvum”, have been reported in asymptomatic and symptomatic humans in England [29], South Africa [30], the USA [31,32], Australia [33], Mongolia [34], China [19] and Japan [35]. People that test positive for haemoplasmas often report close contact with a range of animal species [36], for example pigs [19], bats [33], dogs [30], cats [30], kangaroos [29,33], birds [33], horses [33], sheep [37] and rodents [30,33,37]. However, studies investigating a direct link between haemoplasma prevalence in humans and co-occurring animal species, such as pigs [19], domestic dogs and horses [13], as well as wild and domestic felids [38], are rare and inconclusive.

It is therefore imperative to gain a better understanding of haemoplasma prevalence, diversity, and potential routes of transmission in free-living species that come into contact with humans [7,25]. Synanthropic and invasive *Rattus* species, in particular, live in close association with humans, relying on their infrastructure for shelter and exploiting freely available food sources [39]. Unfortunately, while haemoplasmas have been detected in free-living wildlife and synanthropic species on all continents apart from Antarctica, geographic biases in sampling mean that data on haemoplasma strain diversity and prevalence on continents such as Australia and Africa are lacking [7,25]. Similarly, just as haemoplasmas have been underreported in some geographical regions, research focusing on haemoplasma strain prevalence in some taxa remains scarce [7]. This is particularly true for the order Rodentia, the most globally widespread and diverse order of mammals [16,25].

Rodents belonging to the *Rattus* genus have long been associated with urban, peri-urban, and rural human settlements, where they live in close contact with humans and their commensal animals, thereby increasing the risk of pathogen spillover events [39]. Hemoplasma species, notably “*Candidatus* Mycoplasma haemomuris subsp. ratti”, as well as other undescribed haemoplasma species, have been reported in *Rattus* species from Hungary [23], Brazil [8], Japan [21], and Chile [25]. However, data on *Rattus*-associated haemoplasma prevalence, diversity and mode of transmission remain poorly understood in some geographical regions, specifically on the African continent [7,25]. Currently, there is only one published report of novel haemoplasmas in free-living indigenous subterranean mole-rats (*Bathyergus suillus*, *Fukomys damarensis* and *Cryptomys hottentotus hottentotus*) sampled in the Western Cape Province, South Africa [40], and no haemoplasma data are available for indigenous and invasive species within this country [7,40].

Gauteng Province, the economic hub of South Africa, represents a densely populated region with the highest levels of HIV/TB co-infections in the country, placing immunocompromised individuals at risk of more severe infection should they encounter zoonotic pathogens [41,42]. Within this region, many HIV- and TB-positive patients live in informal rural, urban, and peri-urban settlements, landscapes in which three synanthropic and invasive *Rattus* species, *viz. R. rattus*, *R. norvegicus* and *R. tanezumi* thrive where direct and indirect inter-specific contact is frequent [39]. The distributional range of these three *Rattus* species varies in South Africa [43]. *Rattus Rattus* is predicted to occur mostly in coastal areas in the Western Cape, Eastern Cape, and KwaZulu-Natal Provinces and in urban inland areas in the Gauteng and Free State Provinces of South Africa, *R. norvegicus* is predicted to occur in coastal areas as well as urban inland areas in the Gauteng and Limpopo Provinces of South Africa, and *R. tanezumi* is predicted to occur predominantly inland in areas such as Gauteng and Mpumalanga Provinces and along the northeast and southeast coast of the country, with the distributions of these three *Rattus* species shown to overlap, especially in urban areas, such as in informal settlements in Gauteng Province, where niche partitioning of food resources and shelter likely allows them to co-exist with each other and indigenous species such as *Mastomys* spp. [43].

Furthermore, considering the need to better understand the dynamics of haemoplasmas cycling in free-living species occurring on the African continent, the aim of this study was to investigate the prevalence, diversity, and potential transmission routes of haemoplasmas in three *Rattus* species (*R. rattus*, *R. norvegicus,* and *R. tanezumi*) sampled from Gauteng Province of South Africa using conventional PCR methods. It was predicted that haemoplasmas detected in the three *Rattus* species would be closely related to haemoplasmas previously detected in other *Rattus* species sampled from Japan, Hungary, and Brazil. As *R. norvegicus* is the most aggressive of the three and has been shown to be infected by up to 1.4 times more ectoparasites compared to *R. rattus* [43,44]. It was predicted that *R. norvegicus* would display the highest haemoplasma prevalence of the three *Rattus* species assessed. Furthermore, it was predicted that haemoplasmas would be detected both in buccal swabs and associated ectoparasites collected from the *Rattus* samples screened for haemoplasmas, and that they would likely use more than one concurrent transmission route [7]. Finally, it was predicted that male individuals would likely show a higher haemoplasma prevalence than females and that adult *Rattus* individuals would display a higher haemoplasma prevalence compared to younger individuals.

## 2. Materials and Methods

### 2.1. Sample Collection

**Figure 1:**
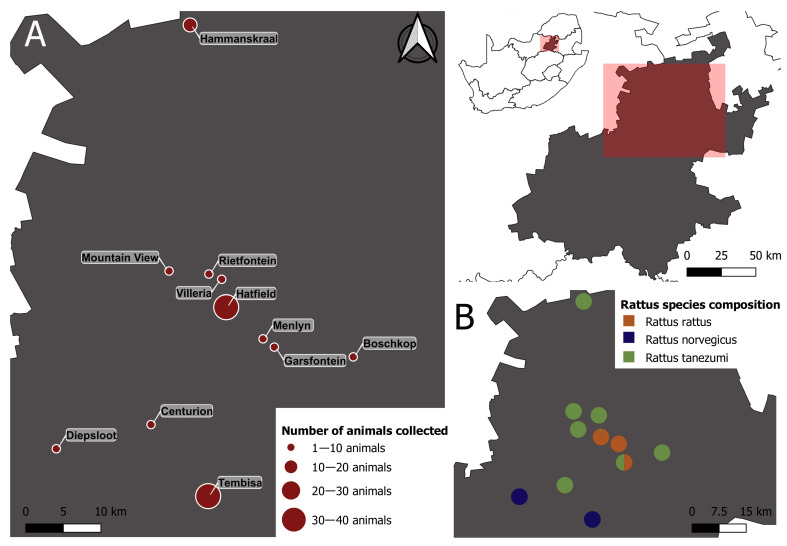
Sampling map of *Rattus* samples collected across 11 different sampling sites across the Gauteng Province. A total of 99 *Rattus* samples comprising of three *Rattus* species, *viz. Rattus rattus* (n = 36), *R. norvegicus* (n = 35), and *R. tanezumi* (n = 28) were collected and screened for haemotropic mycoplasmas. (**A**) indicates the number of *Rattus* samples collected per sampling site while (**B**) indicates the *Rattus* species composition per site.

Rodents belonging to the genus *Rattus* (*R. rattus*, *R. norvegicus,* and *R. tanezumi*) in this study were sampled from in and around office buildings, storage facilities, and formal and informal human dwellings in 11 different localities across Gauteng Province, South Africa (Figure 1; Table 1). These samples were sourced from two prior studies investigating the genetics and/or pathogen diversity and transmission routes of invasive *Rattus* species [39,45].

Samples were obtained under permit number CPF6 0032 from Gauteng Department of Nature Conservation, Johannesburg, South Africa. Permit numbers 13788 and 12/11/1/1(2022MvA) to conduct this study were granted under the terms of Section 20 of the Animal Diseases Act (Act no. 35 of 1984) of the South African Department of Agriculture, Forestry and Fisheries. An animal ethics approval number ECO25-10 and NAS059/2021 to conduct this study was granted by the Animal Ethics Committee of the University of Pretoria, Pretoria, South Africa.

DNA was extracted from buccal swabs collected from 99 rats and stored in 1:1 phosphate-buffered saline (PBS)/glycerol solution and from kidney samples of the corresponding animals. Buccal swabs were selected to test if haemoplasmas could be detected in the saliva of free-living rodent species based on a prior report of haemoplasma presence in saliva of experimentally infected rodents [11]. Kidney samples were selected as (a) they were the only tissue samples available from previous studies, (b) represent a blood-rich organ and (c) kidney samples have tested positive for haemoplasma presence, in some cases up to 11 months after inoculating test animals with haemoplasma-infected blood [46,47]. The Roche High Pure PCR Template Preparation Kit (Roche Diagnostics, Johannesburg, South Africa) was used to prepare the DNA extracts made available for this study (Table S1).

In addition, DNA extracts of 47 individual ectoparasites collected from 18 rats were included in this study. Ectoparasites were individually removed by combing the fur of Rattus specimens in a biological safety hood. All ectoparasites collected were preserved in 100% ethanol and subsequently sorted into mites, ticks, lice, and fleas for further identification. Ectoparasites were removed from 100% ethanol and rinsed three times with double distilled water. Whole ectoparasites were individually crushed using sterile sand and a pestle and eluted in 200 μL of PBS. Genomic DNA was extracted from ectoparasite homogenates of ticks using the High Pure PCR Template Preparation Kit (Roche Diagnostics, Johannesburg, South Africa), according to the manufacturer’s protocol, with the exception that ectoparasite DNA was eluted in 100 μL instead of 200 μL. All extracts were stored at −20 °C until further processing.

### 2.2. Rattus Age Classes

Based on the level of maxillary molar toothrow cusp eruption and wear [48], samples belonging to the three *Rattus* species were grouped into five relative age classes (tooth-wear class I–V). Following the methods of others [39,49], these tooth-wear classes were further divided, where class I represented juvenile individuals, age classes II and III represented sub-adults, and age classes IV and V represented adults.

### 2.3. Rattus and Ectoparasite Species Identification

Species identification of all *Rattus* samples was confirmed through the amplification of the cytochrome *b* (cyt *b*) gene region as part of a prior study [39]. The ectoparasites used in the current study were identified by using published morphological keys for fleas [50,51,52,53], ticks [54,55,56,57,58,59] and mites [60,61,62]. Fleas and ticks were identified to species level while mites were identified to genus level.

### 2.4. Haemoplasma Screening

#### 2.4.1. 16S rRNA PCR Assays

Using methods described previously [40], an initial screening of all DNA extracts from buccal swabs and kidney samples was conducted by amplifying an ~300 bp fragment of the 16S rRNA gene region using the primer combination MyChlos-1F and Mycop-1R (Table 2). Subsequently, amplification of a larger fragment of the 16S rRNA gene region was attempted for all sequence-confirmed positive haemoplasma variants using two additional primer sets, Myco16S-322s/HemMycop16S-1420as (Table 2) [32] and 27F/ Mycop-1R (Table 2) [40,63]. All ectoparasite samples were screened using both the MyChlos-1F/Mycop-1R primer set [40] as well as the Myco16S-322s/HemMycop16S-1420as primer set [32].

#### 2.4.2. Additional Gene Regions 

A subset of sequence-confirmed haemoplasma positive samples (n = 10), which were representative of all haemoplasma variants recovered during the initial 16S rRNA screening phase, were selected for amplification with additional published primer sets targeting the Rnase P, *dna*K, and *gap*A gene regions (Table 2).

#### 2.4.3. Polymerase Chain Reaction (PCR) Amplification and Nucleotide Sequencing

PCRs were performed in a final reaction volume of 40 µL, containing a final concentration of 1 × Dream *Taq* Buffer, 0.2 µM dNTPs (Fermentas), 1.5 U of Dream *Taq* (Thermo Fisher Scientific, Waltham, MA, USA), 0.4 µM of each primer, and 3 µL of template DNA. Touchdown PCRs with an initial denaturation at 96 °C for 12 s, primer annealing for 30 s at annealing temperatures optimised for each assay (Table 2), elongation at 70 °C with variable time (depending on the size of amplicon targeted) and final elongation at 70 °C for 1 min, were performed on the same ABI 2720 thermal cycler (Applied Biosystems, Foster City, CA, USA)). The PCR products were separated by 1.5% agarose gel electrophoresis and size was estimated against a DNA molecular weight marker (Fermentas, Waltham, MA, USA). All products of the correct size were purified directly from the tube using the Roche High Pure PCR product purification kit (Roche Diagnostics GmbH, Mannheim, Germany) and supplier-prescribed protocols.

Purified DNA was cycle sequenced using BigDye Terminator Cycle Sequencing Ready Reaction Kit (Applied Biosystems, Foster City, CA, USA). Sequence chromatograms viewed in the Chromas programme in MEGA version 6 [64] were edited and aligned to generate contiguous sequences and used in nucleotide BLAST (BlastN) searches against the GenBank database (www.ncbi.nlm.nih.gov/blast), accessed on 10 October 2021) to identify the closest sequence matches [65].

### 2.5. Phylogenetic Analyses

Both the 16S rRNA and Rnase P nucleotide sequences generated through screening *Rattus* kidney samples in this study were complemented with reference sequences and aligned with ClustalW in Mega 6 [64]. For the 16S rRNA dataset, *Mycoplasma fastidiosum*, a closely related sister taxon to the haemotropic *Mycoplasma* species lineage, was included as an outgroup. For the Rnase P gene region, *Mycoplasma fastidiosum* and *Mycoplasma leachii* were included as outgroups. The final aligned 16S rRNA and Rnase P datasets were used to identify the best-fit model of sequence evolution under the Bayesian Information Criterion (BIC) in Mega 6 [64]. For the 16S rRNA gene region, the best-fit model of sequence evolution was the Tamura-Nei model, while the best-fit model of sequence evolution was the Tamura 3-parameter model for the Rnase P gene region. These models of sequence evolution were used for Minimum Evolution (ME) and Maximum Likelihood (ML) analyses performed in Mega 6 [66] and phyML [67], respectively. Nodal support was evaluated through 10,000 non-parametric bootstrap replications. The best-fit models guided the selection of priors for Bayesian Inference (BI) performed in MrBayes [68,69]. For both the 16S rRNA and the Rnase P BI analyses, trees were sampled every 100th iteration, and MCMC Trace Analysis Tool version 1.6.0 [70] was used to confirm a 25% burn-in.

### 2.6. Statistical Analyses

Chi-square (*χ*^2^) test was used to determine differences in haemoplasma prevalence between host species, sex, and age. Multi-comparison Fisher’s exact test with a Bonferroni correction was used post hoc to find which host species had significantly different haemoplamsa prevalence. Fisher’s exact test was used to determine differences in haemoplasma prevalence between host sex per *Rattus* species. Statistical analyses were performed using algorithms in the statistical program R with the use of R Studio [71].

## 3. Results

### 3.1. Haemoplasma Prevalence in Ectoparasites

Of the 47 ectoparasites screened, only one of the nine positive extracts produced a 120 nucleotide (nt) stretch of unambiguous sequence with the MyChlo-F/Mycop-R primer set (sequence-confirmed haemoplasma prevalence of 2.1%). All other sequences are comprised of mixtures. Nucleotide BlastN searches confirmed that the 120 nt fragment had a 94.1% sequence identity to a haemoplasma sequence (Genbank accession number: MK295631), which has previously been detected in the small big-eared brown bat (*Histiotus montanus*) (Figure S1). Of the 47 ectoparasites screened with the Myco16S-322s/HemMycop16S-1420as primer set, 13 produced amplicons of the correct size and were cycle sequenced, with BlastN searches revealing that the closest matches for seven samples were to non-target bacterial genera (Table S2).

### 3.2. Haemoplasma Prevalence in Buccal Swabs

Of the 99 *Rattus* buccal swabs screened, one sample of the 14 positive amplicons detected produced a clean haemoplasma sequence during the initial 16S rRNA screening (sequence-confirmed haemoplasma prevalence of 1.0%). The haemoplasma genome presence in this buccal swab sample from an *R. tanezumi* individual was confirmed through two separate PCR amplifications and nucleotide sequencing events with the MyClos-1F/Mycop-1R primer set. Similarly, the corresponding kidney sample of this individual was confirmed to be haemoplasma-positive through nucleotide sequencing and to be identical to the 16S rRNA gene fragment detected in the buccal swab. Despite multiple attempts, it was not possible to generate a larger 16S rRNA fragment or generate sequence data for alternative gene regions for this haemoplasma-positive buccal swab.

### 3.3. Haemoplasma Prevalence in Rattus Kidneys

**Figure 2:**
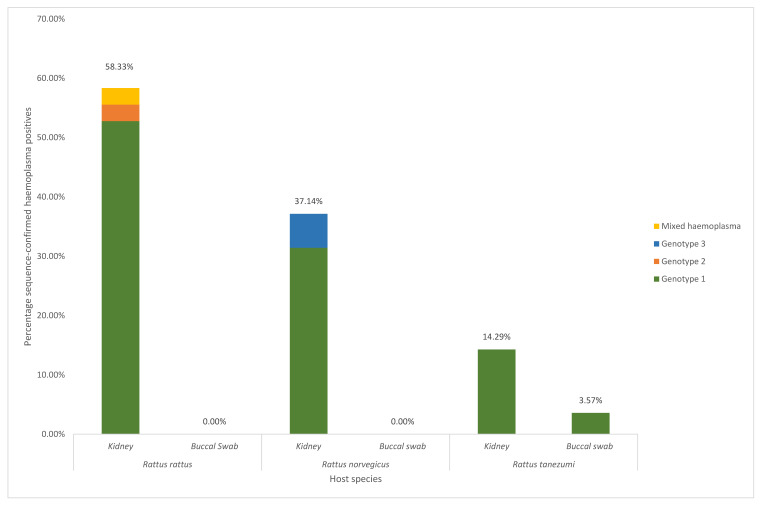
Percentage of sequence-confirmed haemoplasma positive samples detected in kidney and buccal swabs of 99 *Rattus* individuals, comprising of three species (*Rattus rattus*, *R. norvegicus,* and *R. tanesumi*) assessed in this study. In total, three different haemoplasma genotypes and one mixed haemoplasma sequence were detected in the *Rattus* samples screened. Genotype prevalence per tissue type and per *Rattus* species are indicated.

Of the 99 *Rattus* kidney samples screened, 38 of the positive amplicons detected produced unambiguous haemoplasma 16S rRNA sequences, corresponding to an overall sequence-confirmed haemoplasma prevalence of 38.4% in all *Rattus* kidney samples screened. In total, three distinct haemoplasma genotypes were detected in the three *Rattus* species screened. Genotype 1, which was detected in 21 *R. rattus* kidney samples (58.3%), 13 *R. norvegicus* kidney samples (37.1%), and four *R. tanezumi* kidney samples (14.3%), was the most prevalent genotype, occurring at an overall prevalence of 34.3%, followed by genotype 3 (overall prevalence of 2.0%), which was detected in two *R. norvegicus* samples, and genotype 2 (overall prevalence of 1.0%), which was identified from a single *R. rattus* kidney sample (Figure 2).

### 3.4. 16S rRNA Nucleotide Searches and Phylogenetic Analyses

**Figure 3:**
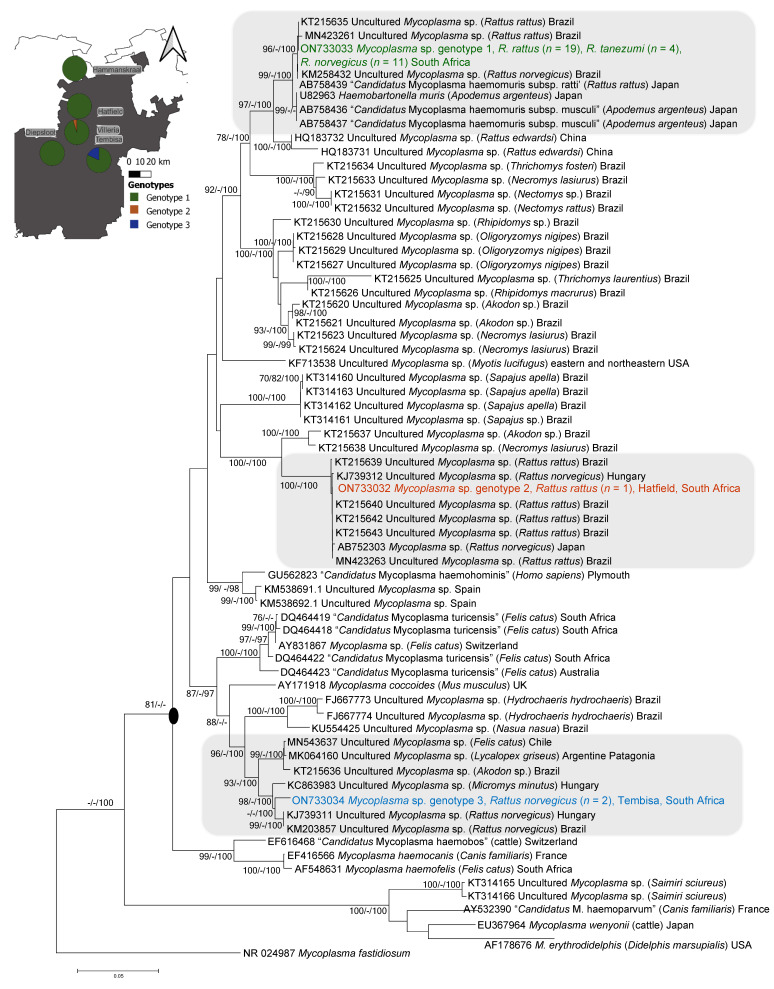
Minimum Evolution tree depicting genetic relationships of the three *Rattus*-associated hemoplasma genotypes detected in three *Rattus* species, *viz. Rattus rattus*, *R. norvegicus,* and *R. tanezumi*, sampled across the Gauteng Province of South Africa, with relevant reference sequences. Genbank accession numbers of the three haemoplasmas detected in the current study are indicated. –Genotype 1, which formed a well-supported clade with *M. haemomuris*, is indicated in green, Genotype 2 is indicated in orange and Genotype 3 is indicated in blue. The three haemoplasma genotypes detected in this study fell within the Haemofelis cluster (circled at the node) and formed three, separate well-supported clades with haemoplasmas strains previously detected in *Rattus* species (highlighted in grey). The tree was inferred using the aligned 1063 nucleotide data set of the 16S rRNA gene region. Bootstrap support values ≥70% from the minimum evolution analysis (ME; 10,000 bootstrap replicates), maximum likelihood (ML; 10,000 bootstrap replicates); posterior probability support values ≥90% from the Bayesian inference (BI; 1,000,000 generations sampled every 10 generations with a 25% burn-in) are indicated as ME/ML/BI next to each node.

Through amplification and sequencing using primer sets Myco16S-322s/HemMycop16S-1420as and 27F/Mycop-1R, contiguous 16S rRNA sequences, 1063 nt in length, were generated for all three haemoplasma genotypes detected in kidney samples, based on the initial 16S rRNA screening with the MyChlo-1F/Mycop-1R primer set. These were complemented with reference sequences obtained from the Genbank database and resulted in a final aligned 16S rRNA dataset, 1063 nt in length and comprising 67 taxa. Phylogenetic analyses revealed that all three *Rattus*-associated haemoplasmas detected in the current study were phylogenetically distinct from each other and fell into three separate, well-supported clusters (Figure 3). Nucleotide BlastN searches revealed that Genotype 1, which was detected in all three *Rattus* species, showed 100% sequence identity to a haemoplasma sequence previously detected in *R. rattus* sampled from Brazil (KT215635). The 16S rRNA phylogeny further confirmed that Genotype 1 clustered with *Mycoplasma haemomuris* strains detected in *R. rattus* from Brazil and Japan (96–100% nodal support; Figure 3).

Genotype 1 also formed a well-supported sister clade to the subspecies “*Candidatus* Mycoplasma subsp. musculi” (sequence identity of 99.4% to U82963; AB758436 and AB75837), previously detected in *Apodemus argenteus* in Japan. Phylogenetic analyses and nucleotide BlastN searches showed that Genotype 2 formed a well-supported cluster with novel haemoplasma sequences previously detected in *Rattus* species from Brazil (100% sequence identity to MN423263, KT215643, KT215640 and KT215642) and was closely related to strains from Hungary (99.8% sequence identity to a haemoplasma detected in *R. norvegicus*, KJ739312) and Japan (99.5% sequence identity to a haemoplasma detected in *R. norvegicus*, AB752303) (Figure 3). Genotype 3 formed a well-supported cluster with haemoplasma sequences previously detected in *R. norvegicus* samples from Hungary (97.8% sequence identity to KJ739311) and Brazil (97.8% sequence identity to KM203857). Together, these *Rattus-*associated haemoplasma strains formed a lineage sister to a novel haemoplasma sequence previously detected in *Micromys minutus* from Hungary (97.7% sequence identity to KC863983). All 16S rRNA gene sequences generated in this study were submitted to Genbank under accession numbers ON733032–ON733034.

### 3.5. Alternative Gene Regions

**Figure 4:**
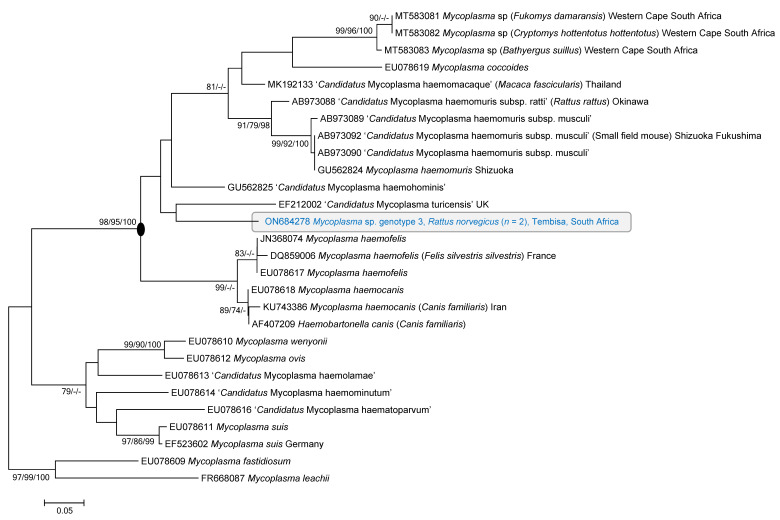
Minimum evolution tree depicting genetic relationships of a *Rattus*-associated hemoplasma genotype, indicated in blue, detected in a *R. norvegicus* sampled in the Gauteng Province of South Africa, with relevant reference sequences. Genbank accession numbers of the three haemoplasmas detected in the current study are indicated. This haemoplasma genotype fell within the Haemofelis cluster (circled at the node), but close phylogenetic relationships could not be resolved. The tree was inferred using the aligned 225 nucleotide data set of the Rnase P gene region. Bootstrap support values ≥70% from the minimum evolution analysis (ME; 10,000 bootstrap replicates), maximum likelihood (ML; 10,000 bootstrap replicates); posterior probability support values ≥90% from the Bayesian inference (BI; 1,000,000 generations sampled every 10 generations with a 25% burn-in) are indicated as ME/ML/BI next to each node.

Despite multiple attempts, including varying the annealing temperatures (Table 1) and extension times, as well as trying multiple primer combinations (Table 1), none of the samples confirmed positive by 16S rRNA screening, amplified with primer sets targeting the *dna*K and *gap*A gene regions. Similarly, despite multiple attempts, only one positive *R. norvegicus* kidney sample was amplified with the Rnase P gene primers, with all other samples screened either failing to amplify, producing mixed amplicons, or showing non-specific amplification (Table S3). The Rnase P sequence was generated for a sample identified as genotype 3 by the 16S rRNA gene analysis (Figure 3 and Figure 4). The final aligned Rnase P dataset consisted of 28 taxa and had a total length of 225 nt. Phylogenetic analyses showed that this sequence fell within the haemofelis cluster with a high level of support (98%, 95%, and 100 nodal support for ME, ML, and BI, respectively) but could not resolve close phylogenetic relationships between the detected sequence and reference haemoplasma sequences obtained from the Genbank database (Figure 4). Nucleotide BlastN searches indicated that the sequence detected had the highest sequence match (84.5%) to ‘*Candidatus* Mycoplasma haemohominis’ (Genbank accession number: GU562825). The RnaseP gene sequence generated in this study was submitted to Genbank under accession number ON684278.

### 3.6. Statistical Analyses

For the kidney samples, a statistically significant difference in haemoplasma prevalence was found between the host species assessed (χ^2^ = 12.96; df = 2; n = 99; *p* < 0.05), with *R. rattus* showing the highest haemoplasma prevalence (58.3%), followed by *R. norvegicus* (37.1%) and finally *R. tanezumi* (14.3%) (Figure 2). *Rattus rattus* had a significantly higher haemoplasma infection rate compared to *R. tanezumi* (*p* < 0.05). There was no significant difference in haemoplasma prevalence between *R. norvegicus* and *R. rattus* (*p* = 0.29) or between *R. norvegicus* and *R. tanezumi* (*p =* 0.15). In contrast, no statistically significant difference was found between host sex (χ^2^ = 3.59 × 10^−31^; df = 1; n = 99; *p* = 1.00), with overall haemoplasma PCR-positivity being 40.7% for males and 35% for females or between host age (χ^2^ = 4.28; df = 2; n = 99; *p* = 0.12), with overall haemoplasma PCR-positivity being 21.0% for juveniles, 35.3% for subadults and 47.8% for adults. In addition, differences in haemoplasma prevalence by host sex were not statistically significantly different when assessed by Rattus species (*R. rattus*: odds ratio = 1.24, 95% CI = 0.23–6.51, n = 36, *p* = 1.00; *R. norvegicus*: odds ratio = 1.16, 95% CI = 0.24–5.75, n = 35, *p* = 1.00; *R. tanezumi*: odds ratio = 0.72, 95% CI = 0.05–11.56, n = 28, *p* = 1.00). However, the statistical results need to be viewed in light of the relatively small sample size (n = 99).

## 4. Discussion

Haemoplasmas have been detected in a diverse range of mammalian species, including species of artiodactyls, carnivores, primates, marsupials, chiroptera, and rodents [7]. Haemoplasmas vary from being asymptomatic in some hosts, to causing potentially fatal haemoplasmosis or haemolytic anaemia in other hosts, such as domestic cats, dogs, and humans [7]. However, data on hemoplasma prevalence in free-living species worldwide and an understanding of the potential routes of hemoplasma transmission are limited [2,72]. This study represents the first report of haemoplasmas cycling in free-living *Rattus* species sampled from South Africa. Rodents are well-known carriers of various pathogens, including haemoplasmas, which have been reported in diverse rodent species sampled from Hungary, Brazil, Japan, Israel, Chile, and South Africa [8,11,21,23,25,40]. The haemoplasma prevalence of 38.4% found in the current study falls within the range of haemoplasma prevalence reported previously in free-living *Rattus* species sampled from Japan (overall prevalence of 11.1% detected in *R. norvegicus* [21]), Hungary (overall prevalence of 92.9%, detected in *R. norvegicus* [23]), and Brazil (overall prevalence of 63.5%, detected in *R. norvegicus* [8], the overall prevalence of 46.2% detected in *R. rattus* [22] and the overall prevalence of 30.7% detected in *R. rattus* [16]). However, it should be noted that these previous studies screened for haemoplasma presence using DNA extracted either from blood [8,21] or spleen samples [16,22,23], while the current study used DNA extracted from kidney samples. Whilst this may have influenced the overall haemoplasma prevalence detected [7,46,47], it nevertheless serves to confirm haemoplasma presence and diversity in *Rattus* species sampled in the Gauteng Province of South Africa.

The observation that the three haemoplasma genotypes detected in the current study fell within the haemofelis cluster with high levels of support is in accordance with other *Rattus-*associated strains detected previously [8,16,21,22,23,25]. Currently, only two rodent-associated haemoplasmas have been described, namely, *Mycoplasma coccoides* and *M. haemomuris*; however, in the last decade, novel haemoplasmas have been detected in a wide range of rodent species worldwide [7,25,73]. Moreover, while rodent-associated haemoplasmas are generally considered to be host-specific, a growing body of evidence suggests that rodent-associated haemoplasmas are capable of host switching and may hold zoonotic potential, making it imperative to understand the dynamics of haemoplasma cycling in various rodent species [15,25,26]. All three genotypes detected in the current study formed well-supported clades with haemoplasmas previously detected in *Rattus* species (Figure 3), supporting our prediction that the haemoplasmas detected by the current study would be phylogenetically similar to haemoplasmas previously detected in *Rattus* species. These results also support, in part, the prediction that *Rattus*-associated haemoplasmas are likely restricted to causing infection in closely related *Rattus* species, with unknown barriers expected to prevent haemoplasma transmission between synanthropic *Rattus* species and wildlife occupying the same area [16,22].

Certainly, the inter- and intra- specific routes of haemoplasma transmission remain poorly understood [7,11]. Traditionally, emphasis has been placed on shared ectoparasite vectors as the main route of haemoplasma transmission [22,23]. The current study could only detect one sequence-confirmed haemoplasma-positive in a *Haemaphysalis elliptica* tick, representing the first detection of haemoplasmas in an ectoparasite sampled from South Africa. This tick is a generalist species, associated with a wide range of rodent species during its immature stages, while adults are found on both wild and domestic carnivores [59,74]. However, the fact that the current study could only detect one haemoplasma-positive case in *H. elliptica* collected from a haemoplasma-negative R. tanezumi individual, is in accordance with other studies reporting discrepancies in haemoplasma infection status between ectoparasites and their host species [24,26,75]. Previous studies either report discrepancies in haemoplasma infection status between ectoparasites and their host species, fail to detect haemoplasmas in ectoparasites collected from positive hosts or find no difference in haemoplasma prevalence between ectoparasite-free and ectoparasite-infested hosts [24,26,75]. For these reasons, some studies have suggested that ectoparasites may play a limited role, if any, in haemoplasma transmission [11,16].

In the current study, one buccal swab of a confirmed haemoplasma-positive *R. tanezumi* individual tested positive for a *Mycoplasma haemomuris*-like genotype (Figure 2), lending support to assertions that direct transmission during grooming or aggressive host behaviour may represent an important route of haemoplasma transmission [7,11]. This finding in a wild-caught rat complements a prior report of haemoplasma detection in buccal swabs of rodents (*Gerbillus andersoni*) experimentally infected with a *Mycoplasma haemomuris*-like haemoplasma [11]. Haemoplasma infection is characterised by an acute and a chronic phase [7], with Cohen et al. (2018) detecting a low haemoplasma load in buccal swabs only during peak haemoplasma infection of hosts. This may explain the low haemoplasma prevalence found in *Rattus* buccal swabs compared to the prevalence found in *Rattus* kidney samples in the current study (Figure 2). Whilst no bite wounds were recorded for the rodents assessed, aggressive behaviour by *Rattus norvegicus* towards other sympatric congenerics is well-established [44] and thus a possible route of transmission. These results support the prediction in the current study that there may be more than one concurrent route of haemoplasma transmission. However, future studies are needed to determine whether oral haemoplasma presence in rats is transient and only associated with the bacteremic phase.

It has been suggested that males may display higher levels of haemoplasma prevalence due to more aggressive behaviour, which may facilitate the transfer of saliva from infected hosts into the open wounds of uninfected hosts [7]. However, just as in previous studies investigating haemoplasma prevalence in *Rattus* species [11,21], the current study found no significant difference in haemoplasma prevalence between male and female *Rattus* individuals for all three *Rattus* species assessed, lending support to the idea that sex biases in haemoplasma infection likely vary across mammalian taxa [7]. Therefore, the prediction that there would be a significant difference in haemoplasma prevalence between male and female *Rattus* samples screened was rejected. Similarly, it has been suggested that older individuals of certain mammalian taxa, such as felids and canids, may display a higher haemoplasma prevalence compared to younger individuals [7]; however, just as with other studies on American minks (*Neovison vison*) [76] and domestic dogs [77], the current study found no difference in haemoplasma prevalence between different age classes assessed, thereby rejecting the prediction that older *Rattus* individuals would display a higher haemoplasma prevalence compared to younger individuals.

Phylogenetically similar haemoplasma genotypes have been detected in *Rattus* species sampled from geographically disparate locations (Figure 3), suggesting that *Rattus*-associated haemoplasmas were likely introduced into novel environments, such as Brazil and South Africa, along with their host species during historic species introductions [16,22]. Members belonging to the *Rattus* genus likely reached the African continent primarily through the shipping trade but also overland [78]. Molecular analyses indicate that at least three separate *R. rattus* and two separate *R. norvegicus* introductions occurred in South Africa, and that *R. tanezumi* was likely introduced into this country during a single, more recent event [79]. While these three *Rattus* species have been found to occur in sympatry in urban areas, specifically informal settlements in Gauteng Province of South Africa, they vary in behaviour and likely exhibit niche partitioning in the urban environments where they co-exist [43].

*Rattus norvegicus* is an aggressive, mostly ground-dwelling species, which has been found to be infected with up to 1.4 times more ectoparasites compared to *R. rattus*, a less aggressive species, which is an adept climber, closely associated with urban environments, preferring upper floors and rooftops, which may limit its exposure to ectoparasites, such as ticks, which wait for their hosts on the ground [43,44]. *Rattus tanezumi*, which forms part of the *R. rattus* species complex and is known to hybridise with *R. rattus* and may outcompete the other two *Rattus* species with scent marking [43,44,80]. As is the case for *R. rattus*, this species is arboreal but also lives in and around human dwellings, agricultural lands, and natural sites [43,81].

We found that more than one haemoplasma genotype was present in both *R. norvegicus* and *R. rattus*, *viz*. genotypes 1 and 3, and genotypes 1 and 2, respectively, while only one haemoplasma genotype (genotype 1) was detected in *R. tanezumi* (Figure 2 and Figure 3); however, the latter species also had the lowest infection rate. It should be noted that the *R. norvegicus* samples were collected from separate sampling sites compared to *R. rattus* and *R. tanezumi* (Figure 1), which may, in part, restrict the transmission of genotypes 2 and 3 between the different host species. Nevertheless, it is of interest that the current study found that *R. rattus*, which is both less aggressive and displays lower ectoparasite loads compared to *R. norvegicus* [44], had the highest haemoplasma prevalence, thereby rejecting the prediction that *R. norvegicus* would display the highest haemoplasma prevalence, lending credence to doubts regarding the importance of vector-borne transmission.

Importantly, the results of the current study also expand on species-specific differences reported for other pathogenic bacterial genera cycling within the three *Rattus* species in Gauteng Province metropoles. Julius et al. (2021) found a high overall *Streptobacillus* prevalence, with specific *Streptobacillus* species being associated with *R. rattus/R. tanezumi* hosts or *R. norvegicus* hosts, but not both, which is in contrast to the results of the current study that showed that genotype 1 was present in all three *Rattus* species sampled (Figure 3). Moseley et al. (2020) found a high prevalence of *Leptospira borgpetersenii* (overall prevalence of 44%) cycling in, primarily *R. norvegicus* and one *R. rattus* individual within this geographical region [82]. Together, these studies confirm the presence of a broad range of potentially zoonotic bacterial pathogens cycling in *Rattus* species within Gauteng Province [80]. Moreover, the significantly lower levels of haemoplasma prevalence found in *R. tanezumi* compared to *R. rattus* is in accordance with what has been found when assessing broad-range pathogenic bacteria [83] as well as *Streptobacillus* [39]. In *Rattus* species sampled from the same geographical area, Julius et al. (2021) found an almost two-fold higher *Streptobacillus* prevalence in *R. rattus* compared to *R. tanezumi*, whilst in the current study, *R. rattus* had a haemoplasma prevalence of almost four times higher than *R. tanezumi* (Figure 2). As *R. tanezumi* belongs to the same species complex as *R. rattus* [39] and given that in the current study, *R. rattus* and *R. tanezumi* were sampled in close proximity to each other (Figure 1), the results of the current study further support the suggestion by Julius et al. (2021) that there may be unknown genetic or behavioural factors, such as varying levels of aggressive behaviour or ectoparasite load, which may contribute to differences in pathogenic bacterial prevalence between these two morphologically indistinguishable host species.

Both phylogenetic analyses and nucleotide BlastN searches confirmed that the genotype detected in all three *Rattus* species (genotype 1) is closely related to *M. haemomuris*, which has been detected in *R. rattus* from Japan, Hungary, and Brazil [8,21,23]. Moreover, the relatively high prevalence of 38.4% in all samples screened indicates that, just as in other countries, this genotype is likely widespread in free-living *Rattus* species occurring within the Gauteng Province metropoles of South Africa, as this genotype was detected in *Rattus* species sampled from five of the 11 sampling localities (Figure 3). *Mycoplasma haemomuris* has been divided into two subgroups, *viz.* “*Candidatus* Mycoplasma haemomuris subsp. ratti” and “*Candidatus* Mycoplasma haemomuris subsp. musculi” [21,73]. This division is based on genetic differences in the 16S rRNA, 16S-23S rRNA intergenic spacer (ITS), Rnase P, and *dna*k gene regions, with “*Candidatus* Mycoplasma haemomuris subsp. ratti” associated with *Rattus* species sampled from Japan, Hungary, and Brazil, while “*Candidatus* Mycoplasma haemomuris subsp. musculi” is associated with wild mice sampled across Japan (Figure 3) [21,73]. *Mycoplasma haemomuris* is not considered to cause clear clinical symptoms in infected host species [8].

However, in addition to *M. haemomuris*, two additional haemoplasma genotypes were also detected in the *Rattus* kidney samples screened (Figure 3). Genotype 2, detected in one *R. rattus* individual, is identical to *Rattus-*associated haemoplasmas detected in Brazil, Hungary, and Japan (Figure 3), with the authors of prior studies suggesting that this genotype likely represents an undescribed *Rattus*-associated haemoplasma species [16,21,22,23]. Furthermore, in contrast to Genotypes 1 and 2, which showed 100% identities to haemoplasmas detected previously, phylogenetic analyses and nucleotide BlastN searches revealed that Genotype 3, detected in two *R. norvegicus* individuals, represents a novel haemoplasma genotype (Figure 3). This novel genotype was most closely related to a haemoplasma genotype detected in *R. norvegicus* individuals sampled from Brazil [8] and Hungary [23]. Phylogenetic analyses of the 16S rRNA gene region indicate that the *Rattus-*associated genotypes are closely related to haemoplasmas detected in wild rodents, domestic cats, and grey foxes (Figure 3), suggesting that, whilst rare, cross-species transmission of *Rattus*-associated haemoplasmas occurred at some point. This again emphasises the need to better understand the inter- and intra- specific routes of haemoplasma transmission in wild and synanthropic rodent species [7,8].

Based on its phylogenetic distance from *Mycoplasma haemomuris*, Conrado et al. (2015) suggest that the clade within which genotype 3 falls (Figure 3) may represent a novel haemoplasma species, but that the generation of additional gene regions is needed to confirm species status. While the PCR-assays targeting the *dna*K and *gap*A gene regions failed to produce any amplicons in the current study, we were able to successfully generate a single Rnase P sequence for a genotype 3 strain detected in the kidney sample of an *R. norvegicus* individual (Figure 4). Both nucleotide BlastN searches and phylogenetic analyses for this gene region showed that, currently, no close sequence matches are available for this genotype for the Rnase P gene region (Figure 4). While the 16S rRNA gene region is widely used in the identification of unculturable microorganisms and currently forms the bulk of the genetic data available for haemoplasmas on online databases [7,8], these results emphasise the need for data on phylogenetically more informative gene regions such as Rnase P, *dna*k, and *gap*A for haemoplasmas. This is currently constrained by the lack of sensitive haemoplasma-specific PCR assays for alternative gene regions. This was underscored by the fact that we were only able to generate Rnase P data for one of the three genotypes detected. Other authors have reported similar difficulties in generating sequence data for alternative gene regions for rodent-associated haemoplasma strains [22]. 

## 5. Conclusions

This study is the first to report haemoplasma prevalence and diversity in three invasive, wild-caught *Rattus* species from South Africa. While blood and spleen samples and not kidney samples have been traditionally used when testing for haemoplasma prevalence [7], the haemoplasma prevalence of 38.4% found in kidney samples from free-living synanthropic *Rattus* species within the Gauteng Province provides an initial estimate of haemoplasma prevalence in this region and indicates that haemoplasmas are likely widespread in *Rattus* species within the Gauteng Province of South Africa. The sequence-confirmed presence of haemoplasma in a buccal swab provides support for the prediction that haemoplasmas may be transmitted through direct host-to-host contact during social or aggressive interactions [11] and, to our knowledge, is the first time haemoplasmas have been detected in the saliva of a free-living rodent species. While the haemoplasma genotypes detected here were likely introduced into South Africa during the historic introduction of *Rattus* species, phylogenetic analyses suggest that under certain circumstances, historical haemoplasma host-switching events may have occurred [8]. As the three *Rattus* species overlap in distribution, especially in urban areas, and likely have a much wider distribution within South Africa than previously reported [43], *Rattus-*associated haemoplasmas may be widespread in this country and require further investigation. Furthermore, the findings of the current study highlight the need for future studies to further investigate the potential routes of intra- and inter-specific haemoplasma transmission in both free-living synanthropic and wildlife species. Future studies should focus on screening different tissue types (including blood, spleen, lungs, and kidney) to identify the optimal blood-rich sample type for haemoplasma detection. In addition, the evaluation of foetal samples, ectoparasites as well as oral and rectal swabs and environmental samples would permit the assessment of the importance of multiple routes of transmission (vertical, vector, environmental) and their relative importance. The difficulties in generating sequence data for gene regions other than the 16S rRNA gene region highlight the need for the development of sensitive haemoplasma-specific PCR assays targeting alternative non-ribosomal gene regions for the accurate phylogenetic placement of novel haemoplasma genotypes.

## Figures and Tables

**Table 1:** Number of each *Rattus* species (*Rattus rattus*, *R. norvegicus* and *R. tanezumi*) sampled at 11 sampling localities across the Gauteng Province.

Sampling Locality	*Rattus rattus*	*Rattus norvegicus*	*Rattus tanezumi*	Total Animals
Hammanskraal	0	0	12	12
Hatfield	33	0	1	34
Tembisa	0	32	0	32
Villeria	0	0	1	1
Centurion	0	0	1	1
Mountain View	0	0	6	6
Diepsloot	0	3	0	3
Garsfontein	1	0	1	2
Menlyn	2	0	0	2
Boschkop	0	0	5	5
Rietfontein	0	0	1	1
Total	36	35	28	99

**Table 2:** PCR assays used to screen *Rattus* kidney, buccal swabs, and associated ectoparasites for haemotropic *Mycoplasma*.

Primer Set Used (from 5′ to 3′) and Orientation (F:Forwards/R:Reverse)	Reference	Gene Region Targeted	Expected Ampilcon Size (bp)	Ta Used in This Study
Myco16S-322s: GCC CAT ATT CCT ACG GGA AGC AGC AGT (F)	[32]	16S rRNA	~1000	68 °C
HemMycop16S-1420as: GTT TGA CGG GCG GTG TGT ACA AGA CC (R)	[32]			
MyChlo-1F: TGC CAG CAG CTG CGG TAA TAC (F)	[40]	16S rRNA	~300	69 °C
Mycop-1R: CGT TTA CGG TGT GGA CTA CTG (R)	[40]			
27F: AGA GTT TGA TCC TGG CTC AG (F)	[63]	16S rRNA	~700	61 °C
Mycop-1R: CGT TTA CGG TGT GGA CTA CTG (R)	[40]			
RNasePFor1: CTGC GATGGTCGTAATGTTG (F)	[12]	RnaseP	~180	46 °C
RNasePRev1: GAG GAG TTT ACC GCG TTT CA (R)	[12]			
RNasePFor2: TAT TTA AAG TAG AGG AAA GTC (F)	[12]	RnaseP	~210	49 °C
RNasePRev1: GAG GAG TTT ACC GCG TTT CA (R)	[12]			
F34: GACCTAGGTACAACTAACTCYTGTG (F)	[6]	*dna*K	~1055	56 °C; 50 °C
R1139: CCACCTAGTGTTTCAATACTTAGAGTT (R)	[6]			
F34: GACCTAGGTACAACTAACTCYTGTG (F)	[6]	*dna*K	~1288	56 °C; 50 °C
R1367: CCGTTAGCGTCAATAGAGAAGG (R)	[6]			
F34: GACCTAGGTACAACTAACTCYTGTG (F)	[6]	*dna*K	~1720	55 °C; 50 °C
R1802: TTAGTTTTATCTACCTCAGTCTTATCCT (R)	[6]			
F350: GTTATTACTGTTCCAGCATACTTTAA (F)	[6]	*dna*K	~739	53 °C; 50 °C
R1139: CCACCTAGTGTTTCAATACTTAGAGTT (R)	[6]			
F350: GTTATTACTGTTCCAGCATACTTTAA (F)	[6]	*dna*K	~972	53 °C; 50 °C
R1367: CCGTTAGCGTCAATAGAGAAGG (R)	[6]			
F350: GTTATTACTGTTCCAGCATACTTTAA (F)	[6]	*dna*K	~1404	53 °C; 50 °C
R1802: TTAGTTTTATCTACCTCAGTCTTATCCT (R)	[6]			
GAPA-F22: GGATTCGGAAGAATCGGAAG (F)	[6]	*gap*A	~953	52 °C; 50 °C
GAPA-R975: AACAAGCTGATTCACATAAGAAGA (R)	[6]			

## Data Availability

Publicly available datasets were analyzed in this study. These data can be found here: [https://www.ncbi.nlm.nih.gov/genbank/] (accessed on 9 August 2021).

## Supplementary Materials

The following supporting information can be downloaded at: https://www.mdpi.com/article/10.3390/microorganisms10081632/s1, Figure S1. (a) Trimmed 120 nt fragment of the chromatogram generated when screening ectoparasite sampled using the primer set MyClost-F/Mycop-R. The colour lines denote the four nucleotide bases: adenine (A) is indicated in green, cytosine (C) is indicated in blue, guanine (G) is indicated in black and thymine (T) is indicated in red. (b) Alignment result to MK295631 produced when performing a nucleotide blast (BlastN) search for the 120 nt 16S rRNA gene fragment generated when screening a *Haemaphysalis elliptica* tick for haemoplasma prevalence using the MyClost-F/Mycop-R primer set. Table S1: Sample list of *Rattus* species and their associated ectoparasites screened in this study; Table S2: Nucleotide BlastN results obtained when performing nucleotide blast searches against the Genbank database for sequences generated when screening ectoparasites with the Myco-16S-322s/HemMycop16S-1420as primer set. Table S3: PCR results obtained when screening DNA extracted from buccal swabs, kidney samples, and ectoparasite samples using haemoplasma-specific PCR assays targeting the Rnase P gene region [12]. PCR screening and nucleotide sequencing revealed that samples either failed to amplify or produced non-target sequences.

## References

[1] Willi B., Novacco M., Meli M.L., Wolf-Jäckel G.A., Boretti F.S., Wengi N., Lutz H., Hofmann-Lehmann R. (2010). Haemotropic mycoplasmas of cats and dogs: Transmission, diagnosis, prevalence and importance in Europe. Schweiz. Arch. Fur Tierheilkd..

[2] Wang X., Cui Y., Zhang Y., Shi K., Yan Y., Jian F., Zhang L., Wang R., Ning C. (2017). Molecular characterization of hemotropic mycoplasmas (*Mycoplasma ovis* and ’*Candidatus* Mycoplasma haemovis’) in sheep and goats in China. BMC Vet. Res..

[3] Neimark H., Johansson K., Rikihisa Y., Tully J.G. (2001). Proposal to transfer some members of the genera *Haemobartonella* and *Eperythozoon* to the genums *Mycoplasma* with descriptions of ‘*Candidatus* Mycoplasma haemofelis’, ‘*Candidatus* Mycoplasma haemomuris’, ‘*Candidatus* Mycoplasma haemosuis’ and ’*Candidatus* Mycoplasma wenyonii’. Int. J. Syst. Evol. Microbiol..

[4] Neimark H., Peters W., Robinson B.L., Stewart L.B. (2005). Phylogenetic analysis and description of *Eperythrozoon coccoides*, proposal to transfer to the genus *Mycoplasma* as *Mycoplasma coccoides* comb. nov. and request for an opinion. Int. J. Syst. Evol. Microbiol..

[5] Uilenberg G., Thiaucourt F., Jongejan F. (2004). On molecular taxonomy: What is in a name?. Exp. Appl. Acarol..

[6] Hicks C.A.E., Barker E.N., Brady C., Stokes C.R., Helps C.R., Tasker S. (2014). Non-ribosomal phylogenetic exploration of Mollicute species: New insights into haemoplasma taxonomy. Infect. Genet. Evol..

[7] Millán J., Di Cataldo S., Volokhov D.V., Becker D.J. (2021). Worldwide occurrence of haemoplasmas in wildlife: Insights into the patterns of infection, transmission, pathology and zoonotic potential. Transbound. Emerg. Dis..

[8] Conrado F.D.O., Do Nascimento N.C., Dos Santos A.P., Zimpel C.K., Messick J.B., Biondo A.W. (2015). Occurrence and identification of hemotropic mycoplasmas (Hemoplasmas) in free ranging and laboratory rats (*Rattus norvegicus*) from two Brazilian zoos. BMC Vet. Res..

[9] Volokhov D.V., Hwang J., Chizhikov V.E., Danaceau H., Gottdenker N.L. (2017). Prevalence, genotype richness, and coinfection patterns of hemotropic mycoplasmas in raccoons (*Procyon lotor*) on environmentally protected and urbanized barrier islands. Appl. Environ. Microbiol..

[10] Novacco M., Wolf-Jäckel G., Riond B., Hofmann-Lehmann R. (2012). Humoral immune response to a recombinant hemoplasma antigen in experimental ‘Candidatus Mycoplasma turicensis’ infection. Vet. Microbiol..

[11] Cohen C., Shemesh M., Garrido M., Messika I., Einav M., Khokhlova I., Tasker S., Hawlena H. (2018). Haemoplasmas in wild rodents: Routes of transmission and infection dynamics. Mol. Ecol..

[12] Tasker S., Helps C.R., Day M.J., Harbour D.A., Shaw S.E., Harrus S., Baneth G., Lobetti R.G., Malik R., Beaufils J.P. (2004). Phylogenetic analysis of hemoplasma species: An international study. J. Clin. Microbiol..

[13] Vieira R.F.D.C., Vidotto O., Vieira T.S.W.J., Guimaraes A.M.S., Dos Santos A.P., Nascimento N.C., Dos Santos N.J.R., Martins T.F., Labruna M.B., Marcondes M. (2015). Molecular investigation of hemotropic mycoplasmas in human beings, dogs and horses in a rural settlement in southern Brazil. Rev. Inst. Med. Trop. Sao Paulo.

[14] Vergara R.W., Morera Galleguillos F., Jaramillo M.G., Regina N., Almosny P., Arauna Martínez P., Grob Behne P., Acosta-Jamett G., Müller A. (2016). Prevalence, risk factor analysis, and hematological findings of hemoplasma infection in domestic cats from Valdivia, Southern Chile. Comp. Immunol. Microbiol. Infect. Dis..

[15] Di Cataldo S., Kamani J., Cevidanes A., Msheliza E.G., Millán J. (2020). Hemotropic mycoplasmas in bats captured near human settlements in Nigeria. Comp. Immunol. Microbiol. Infect. Dis..

[16] Gonçalves L.R., Herrera H.M., Nantes W.A.G., Santos F.M., Porfírio G.E.D.O., Barreto W.T.G., de Macedo G.C., Assis W.D.O., Campos J.B.V., da Silva T.M.V. (2020). Genetic diversity and lack of molecular evidence for hemoplasma cross-species transmission between wild and synanthropic mammals from Central-Western Brazil. Acta Trop..

[17] Willi B., Tasker S., Boretti F.S., Doherr M.G., Cattori V., Meli M.L., Lobetti R.G., Malik R., Reusch C.E., Lutz H. (2006). Phylogenetic analysis of ‘Candidatus Mycoplasma turicensis’ isolates from pet cats in the United Kingdom, Australia, and South Africa, with analysis of risk factors for infection. J. Clin. Microbiol..

[18] Sykes J.E., Ball L.M., Bailiff N.L., Fry M.M. (2005). ‘*Candidatus* Mycoplasma haematoparvum’, a novel small haemotropic *Mycoplasma* from a dog. Int. J. Syst. Evol. Microbiol..

[19] Yuan C.L., Liang A.B., Yao C.B., Yang Z.B., Zhu J.G., Cui L., Yu F., Zhu N.Y., Yang X.W., Hua X.G. (2009). Prevalence of *Mycoplasma suis* (*Eperythrozoon suis*) infection in and swine-farm workers in Shanghai, China. Am. J. Vet. Res..

[20] Ybañez A.P., Ybañez R.H.D., Armonia R.K.M., Chico J.K.E., Ferraren K.J.V., Tapdasan E.P., Salces C.B., Maurillo B.C.A., Galon E.M.S., Macalanda A.M.C. (2019). First molecular detection of *Mycoplasma wenyonii* and the ectoparasite biodiversity in dairy water buffalo and cattle in Bohol, Philippines. Parasitol. Int..

[21] Sashida H., Sasaoka F., Suzuki J., Watanabe Y., Fujihara M., Nagai K., Kobayashi S., Furuhama K., Harasawa R. (2013). Detection of hemotropic mycoplasmas in free-living brown sewer rats (*Rattus norvegicus*). J. Vet. Med. Sci..

[22] Gonçalves L.R., Roque A.L.R., Matos C.A., Fernandes S.D.J., Olmos I.D.F., Machado R.Z., André M.R. (2015). Diversity and molecular characterization of novel hemoplasmas infecting wild rodents from different Brazilian biomes. Comp. Immunol. Microbiol. Infect. Dis..

[23] Hornok S., Földvári G., Rigó K., Meli M.L., Gönczi E., Répási A., Farkas R., Papp I., Kontschán J., Hofmann-Lehmann R. (2015). Synanthropic rodents and their ectoparasites as carriers of a novel haemoplasma and vector-borne, zoonotic pathogens indoors. Parasites Vectors.

[24] de Sousa K.C.M., Herrera H.M., Secato C.T., Oliveira A.D.V., Santos F.M., Rocha F.L., Barreto W.T.G., Macedo G.C., Pinto P.C.E.D.A., Machado R.Z. (2017). Occurrence and molecular characterization of hemoplasmas in domestic dogs and wild mammals in a Brazilian wetland. Acta Trop..

[25] Alabí A.S., Monti G., Otth C., Sepulveda-García P., Sánchez-Hidalgo M., De Mello V.V.C., Machado R.Z., André M.R., Bittencourt P., Müller A. (2020). Molecular survey and genetic diversity of hemoplasmas in rodents from Chile. Microorganisms.

[26] Sacristán I., Acuña F., Aguilar E., García S., López M.J., Cevidanes A., Cabello J. (2019). Assessing cross-species transmission of hemoplasmas at the wild-domestic felid interface in Chile using genetic and landscape variables analysis. Sci. Rep..

[27] Cabello J., Altet L., Napolitano C., Sastre N., Hidalgo E., Dávila J.A., Millán J. (2013). Survey of infectious agents in the endangered Darwin’s fox (*Lycalopex fulvipes*): High prevalence and diversity of hemotrophic mycoplasmas. Vet. Microbiol..

[28] Volokhov D.V., Becker D.J., Bergner L.M., Camus M.S., Orton R.J., Chizhikov V.E., Altizer S.M., Streicker D.G. (2017). Novel hemotropic mycoplasmas are widespread and genetically diverse in vampire bats. Epidemiol. Infect..

[29] Steer J.A., Tasker S., Barker E.N., Jensen J., Mitchell J., Stocki T., Chalker V.J., Hamon M. (2011). A novel hemotropic *Mycoplasma* (hemoplasma) in a patient with hemolytic anemia and pyrexia. Clin. Infect. Dis..

[30] Maggi R.G., Mascarelli P.E., Havenga L.N., Naidoo V., Breitschwerdt E.B. (2013). Co-infection with *Anaplasma platys, Bartonella henselae* and “*Candidatus* Mycoplasma haematoparvum” in a veterinarian. Parasites Vectors.

[31] Sykes J.E. (2010). Feline hemotropic mycoplasmas. J. Vet. Emerg. Crit. Care.

[32] Maggi R.G., Compton S.M., Trull C.L., Mascarelli P.E., Robert Mozayeni B., Breitschwerdt E.B. (2013). Infection with hemotropic *Mycoplasma* species in patients with or without extensive arthropod or animal contact. J. Clin. Microbiol..

[33] Alcorn K., Gerrard J., Cochrane T., Graham R., Jennison A., Irwin P.J., Barbosa A.D. (2021). First report of “*Candidatus* Mycoplasma haemohominis” infection in Australia causing persistent fever in an animal carer. Clin. Infect. Dis..

[34] Yang D., Xiuzheng T.A.I., Ying Q.I.U., Sheng Y.U.N. (2000). Prevalence of *Eperythrozoon* spp. infection and congenital eperythrozoonosis in humans in Inner Mongolia, China. Epidemiol. Infect..

[35] Hattori N., Kuroda M., Katano H., Takuma T., Ito T., Arai N., Yanai R., Sekizuka T., Ishii S., Miura Y. (2020). “*Candidatus* Mycoplasma haemohominis” in human, Japan. Emerg. Infect. Dis..

[36] Alkan M.L. (2020). Hemoplasma haemohominis, a new human pathogen. Clin. Infect. Dis..

[37] Sykes J.E., Lindsay L.L., Maggi R.G., Breitschwerdt E.B. (2010). Human coinfection with *Bartonella henselae* and two hemotropic mycoplasma variants resembling *Mycoplasma ovis*. J. Clin. Microbiol..

[38] Willi B., Meli M.L., Luthy R., Honegger H., Wengi N., Hoelzle L.E., Reusch C.E., Lutz H., Hofmann-Lehmann R. (2009). Development and application of a universal haemoplasma screening assay based on the SYBR green PCR principle. J. Clin. Microbiol..

[39] Julius R.S., Brettschneider H., Chimimba C.T., Bastos A.D.S. (2021). Zoonotic Disease: Prevalence and Diversity of the Streptobacillus Rat-bite Fever Agent, in Three Invasive, Commensal Rattus Species from South Africa. Yale J. Biol. Med..

[40] Retief L., Bennett N.C., Bastos A.D.S. (2021). Molecular detection and characterization of novel haemotropic *Mycoplasma* in free-living mole rats from South Africa. Infect. Genet. Evol..

[41] Berry K.M., Rodriguez C.A., Berhanu R.H., Ismail N., Mvusi L., Long L., Evans D. (2019). Treatment outcomes among children, adolescents, and adults on treatment for tuberculosis in two metropolitan municipalities in Gauteng Province, South Africa. BMC Public Health.

[42] Motlhale M., Ncayiyana J.R. (2019). Migration status and prevalence of diabetes and hypertension in Gauteng province, South Africa: Effect modification by demographic and socioeconomic characteristics*—*A cross-sectional population-based study. BMJ Open.

[43] Ringani G.V., Julius R.S., Chimimba C.T., Pirk CW W., Zengeya T.A. (2022). Predicting the potential distribution of a previously undetected cryptic invasive synanthropic Asian house rat (*Rattus tanezumi*) in South Africa. J. Urban Ecol..

[44] Brettschneider H., Anguelov R., Chimimba C.T., Bastos A.D.S. (2012). A mathematical epidemiological model of gram-negative Bartonella bacteria: Does differential ectoparasite load fully explain the differences in infection prevalence of *Rattus rattus* and *Rattus norvegicus*?. J. Biol. Dyn..

[45] Lithole A. (2015). Transmission Dynamics of Bartonella in Invasive Rattus from South Africa. Master’s Thesis.

[46] Novacco M., Boretti F.S., Wolf-Jackel G.A., Riond B., Meli M.L., Willi B., Lutz H., Hofmann-Lehmann R. (2011). Chronic ‘‘Candidatus Mycoplasma turicensis’’ infection. Vet. Res..

[47] Novacco M., Riond B., Meli M.L., Grest P., Hofman-lehmann R. (2013). Tissue sequestration of ‘*Candidatus* Mycoplasma turicensis’. Vet. Microbiol..

[48] Chimimba C.T., Dippenaar N.J. (1994). Non-geographic variation in *Aethomys chrysopilus* (De Winton, 1987) and *A. namaquensis* (A Smith 1834) (Rodentia: Muridae) from southern Africa. S. Afr. J. Zool..

[49] Ringani G.V., Zengeya T.A., Pirk CW W., Chimimba C.T. (2022). Assessment of craniometric sexual dimorphism and ontogenetic variation in invasive *Rattus norvegicus* and *R. rattus* from urban and peri-urban areas of Gauteng Province, South Africa. Mammalia.

[50] Ewing H.E. (1943). The Fleas of North America: Classification, Identification and Geographic Distribution of These Injurious and Disease Spreading Insects.

[51] Traub R. (1950). Siphonaptera from Central America and Mexico: A Morphological Study of the Aedeagus with Descriptions of new Genera and Species.

[52] De Meillon B., Davis D.H.S., Hardy F. (1968). Plague in Southern Africa: The Siphonaptera (excluding Ischnopsyllidae).

[53] Segerman J. (1995). Siphonaptera of Southern Africa: Handbook for the Identification of Fleas.

[54] Hoogstraal H. (1958). Notes on African Haemaphysalis ticks. IV. Description of Egyptian populations of the yellow dog-tick, *H. leachii leachii* (Audouin, 1827) (Ixodoidea, Ixodidae). J. Parasitol..

[55] Hoogstraal H. (1964). Notes on African Haemaphysalis ticks. VI. H. spinulosa Neumann, and its relation to biological and nomenclatorial problems in the *H. leachii* group of Africa and Asia (Ixodoidea, Ixodidae). J. Parasitol..

[56] Hoogstraal H., Kanmah K.M. (1972). Notes on African Haemaphysalis tick X.H. (Kaiseriana) Aciculifer Warburton and H. (K) Rugosa santos dias, the African Representatives of the Spinigera subgroup (Ixodoidea, Ixodidae). J. Parasitol..

[57] Pegram R.G., Hoogstraal H., Wassef H. (1981). Ticks (Acari: Ixodoidea) of Ethiopia. I. Distribution, ecology and host relationships of species infesting livestock. Bull. Entomol. Res..

[58] Camicas J.-L., Hervey J.-P., Adam F., Morel P.-C. (1998). The Ticks of the World.

[59] Apanaskevich D.A., Horak I.G., Camicas J.L. (2007). Redescription of *Haemaphysalis (Rhipistoma) elliptica* (Koch, 1844), an old taxon of the Haemaphysalis [*Rhipistoma leachi* group from East and southern Africa, and of *Haemaphysalis (Rhipistoma) leach*i (Audouin, 1826) (Ixodida, Ixodidae). Onderstepoort J. Vet. Res..

[60] Pratt H.D. (1963). Mites of Public Health Importance and Their Control.

[61] Strandtmann R.W., Mitchel C.J. (1963). The Laelaptine mites of the Echino Laelaps complex from the Southwest Pacific area (Acarina: Mesostigmata). Pac. Insects.

[62] Bakker A.S. (1999). Mites and Ticks of Domestic Animals: Identification Guide and Information Source.

[63] Edwards U., Rogall T., Blöcker H., Emde M., Böttger E.C. (1989). Isolation and direct complete nucleotide determination of entire genes. Characterization of a gene coding for 16S ribosomal RNA. Nucleic Acids Res..

[64] Tamura K., Stecher G., Peterson D., Filipski A., Kumar S. (2013). MEGA6: Molecular Evolutionary Genetics Analysis Version 6.0. Mol. Biol. Evol..

[65] Altschul S.F., Gish W., Miller W., Myers E.W., Lipman D.J. (1990). Basic local alignment search tool. J. Mol. Biol..

[66] Nei M., Kumar S. (2000). Molecular Evolution and Phylogenetics.

[67] Guindon S., Gascuel O. (2003). Simple, fast, and accurate algorithm to estimate large phylogenies by maximum likelihood. Syst. Biol..

[68] Huelsenbeck J.P., Ronquist F. (2001). MRBAYES: Bayesian inference of phylogenetic trees. Bioinformatics.

[69] Ronquist F., Huelsenbeck J.P. (2003). MrBayes 3: Bayesian phylogenetic inference under mixed models. Bioinformatics.

[70] Rambaut A., Suchard M.A., Xie W., Drummond A.J. (2014). Tracer v1.6.0 MCMC Trace Analysis Tool. http://beast.bio.ed.ac.uk/Tracer.

[71] RStudio Team (2020). RStudio: Integrated Development for R.

[72] Willi B., Boretti F.S., Meli M.L., Bernasconi M.V., Casati S., Hegglin D., Puorger M., Neimark H., Cattori V., Wengi N. (2007). Real-time PCR investigation of potential vectors, reservoirs, and shedding patterns of feline hemotropic mycoplasmas. Appl. Environ. Microbiol..

[73] Harasawa R., Fujita H., Kadosaka T., Ando S., Rikihisa Y. (2015). Proposal for ‘*Candidatus* Mycoplasma haemomuris subsp. musculi’ in mice, and ‘*Candidatus* Mycoplasma haemomuris subsp. ratti’ in rats. Int. J. Syst. Evol. Microbiol..

[74] Penzhorn B.L., Harrison-White R.F., Stoltsz W.H. (2020). Completing the cycle: *Haemaphysalis elliptica*, the vector of *Babesia rossi*, is the most prevalent tick infesting black-backed jackals (*Canis mesomelas*), an indigenous reservoir host of *B. rossi* in South Africa. Ticks Tick-Borne Dis..

[75] Millán J., Travaini A., Cevidanes A., Sacristán I., Rodríguez A. (2019). Assessing the natural circulation of canine vector-borne pathogens in foxes, ticks and fleas in protected areas of Argentine Patagonia with negligible dog participation. Parasites Wildl..

[76] Sepúlveda-García P., Raffo E., Medina-Vogel G., Muñoz F., Muñoz P., Alabí A., Navarrete-Talloni M.J., Gonçalves L.R., Califre De Mello V.V., Machado R.Z. (2021). Molecular survey of *Bartonella* spp. and haemoplasmas in American minks (*Neovison vison*). Transbound. Emerg. Dis..

[77] Tennant K.V., Barker E.N., Polizopoulou Z., Helps C.R., Tasker S. (2011). Real-time quantitative polymerase chain reaction detection of haemoplasmas in healthy and unhealthy dogs from Central Macedonia, Greece. J. Small Anim. Pract..

[78] Avery D.M. (2021). Hitching a ride: How Black rats (*Rattus rattus*) could have reached southern Africa. Iziko Mus. South Afr. Cape Town South Afr..

[79] Bastos A.D., Nair D., Taylor P.J., Brettschneider H., Kirsten F., Mostert E., Von Maltitz E., Lamb J.M., Van Hooft P., Belmain S.R. (2011). Genetic monitoring detects an overlooked cryptic species and reveals the diversity and distribution of three invasive *Rattus* congeners in south Africa. BMC Genetics.

[80] Guo H.L., Teng H.J., Zhang J.H., Zhang J.X., Zhang Y.H. (2017). Asian house rats may facilitate their invasive success through suppressing brown rats in chronic interaction. Front. Zool..

[81] Wu D.L., Shih H.C., Wang J.K., Teng H.J., Kuo C.C. (2021). Commensal Rodent Habitat Expansion Enhances Arthropod Disease Vectors on a Tropical Volcanic Island. Front. Vet. Sci..

[82] Moseley M., Naidoo K., Bastos A., Retief L., Frean J., Telfer S., Rossouw J. (2020). Multi-locus sequence analyses reveal a clonal *L. borgpetersenii* genotype in a heterogeneous invasive *Rattus* spp. community across the City of Johannesburg, South Africa. Parasites Vectors.

[83] Julius R.S., Bastos A.D., Brettschneider H., Chimimba C.T. Dynamics of Rodent-Borne Zoonotic Diseases and Their Reservoir Hosts: Invasive Rattus in South Africa. Proceedings of the 25th Vertebrate Pest Conference.

