# Supplementary material for: Haemoplasma Prevalence and Diversity in Three Invasive Rattus Species from Gauteng Province, South Africa
Source: Microorganisms. 2022 Aug 12;10(8):1632. doi: 10.3390/microorganisms10081632 (PMC9413481; doi:10.3390/microorganisms10081632)
Supplement: Supplementary file 1 [file microorganisms-10-01632-s001.zip › microorganisms-1843429-supplementary/Figure S1.pdf]

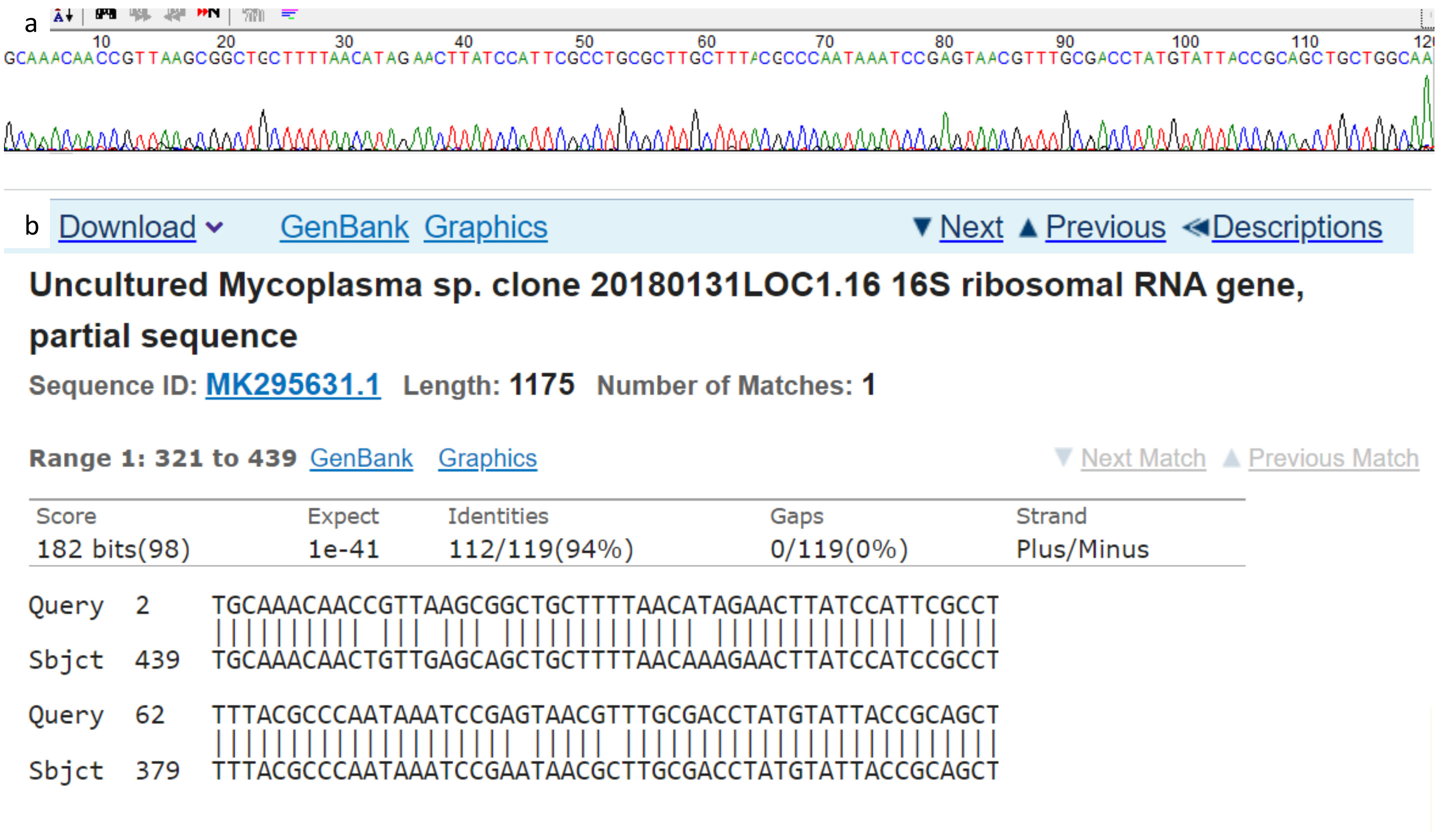

Figure S1. (**a**) Trimmed 120 nt fragment of the chromatogram generated when screening ectoparasite sampled using the primer set MyClost-F/Mycop-R. The colour lines denote the four nucleotide bases: adenine (A) is indicated in green, cytosine (C) is indicated in blue, guanine (G) is indicated in black and thymine (T) is indicated in red. (**b**) Alignment result to MK295631 produced when performing a nucleotide blast (BlastN) search for the 120 nt 16S rRNA gene fragment generated when screening a *Haemaphysalis elliptica* tick for haemoplasma prevalence using the MyClost-F/Mycop-R primer set.
